# High-temperature solvent-free synthesis of low-molecular-weight organogelators consisting of starch-derived 1,5-anhydro-d-glucitol coupled with fatty acids†

**DOI:** 10.1039/d3ra01328f

**Published:** 2023-03-21

**Authors:** Shiro Komba, Rika Iwaura

**Affiliations:** a Food Research Institute, National Agriculture and Food Research Organization 2-1-12, Kannondai Tsukuba Ibaraki 305-8642 Japan skomba@affrc.go.jp

## Abstract

We previously developed low-molecular-weight organogelators that can gel various types of oils by introducing various fatty acids into 1,5-anhydro-d-glucitol (1,5-AG), a cyclic polyol derived from starch. These gelators are composed of ester bonds between naturally occurring materials. We have established a method to synthesize these gelators by heating powdered 1,5-AG and powdered fatty acids to 230 °C to liquefy the compounds without using organic solvents or activated fatty acids and then performing the esterification reaction without a catalyst or with a base catalyst. This has created the possibility of synthesizing these gelling agents at very low cost.

## Abstract 2

We have succeeded in synthesizing organogelators in high yield by simply mixing 1,5-AG derived from corn starch and a free fatty acid derived from natural oils in a reaction vessel and heating to 230 °C without adding any solvent.

## Graphical abstract

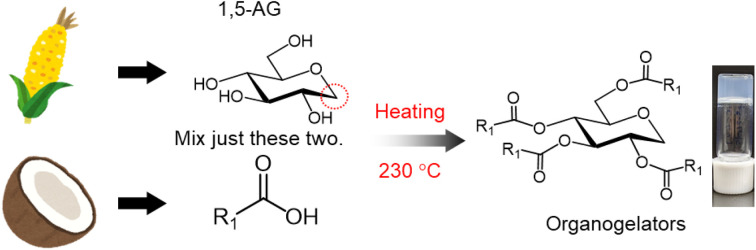

## 1. Introduction

Gelators are compounds that turn liquids into gels. There are two types of gelators, *i.e.*, polymer gelators and low-molecular-weight gelators. Low-molecular-weight gelators form fibers by self-assembly and confine the liquid between the fibers, thereby eliminating the liquid's fluidity and creating a gel.^1–6^ We previously synthesized derivatives of 1,5-anhydro-d-glucitol (1,5-AG), a cyclic polyol obtained by enzymatic and fermentation treatments of starch,^7,8^ containing various linear saturated fatty acids of different lengths *via* ester linkages, and reported that these compounds can gelatinize various organic solvents (C-AG series).^9–11^ We also synthesized compounds in which a linear saturated fatty acid with an amide group was introduced into 1,5-AG *via* ester linkages to further strengthen the intermolecular hydrogen bond (GABA-AG series); as a result, the effect of the amide group produced a harder and more transparent gel than those of the C-AG series without an amide group.^12^ These organogelators have been synthesized by using activated fatty acids in organic solvents. Specifically, the C-AG series was synthesized at 90 °C using expensive fatty acyl chlorides in a pyridine/DMF solvent. The GABA-AG series was synthesized at 60 °C using the expensive condensing agents DIC (*N*,*N′*-diisopropylcarbodiimide) and DMAP (4-dimethylaminopyridine) in 1,2-dichloroethane solvent after synthesis of *N*-fatty acyl-GABA. In the future, the key to mass synthesis at the factory level will be how to reduce the cost of synthesis without using expensive reagents. In particular, the C-AG series is being considered for use as a modifier of asphalt for road paving, and price will be the most important factor in its practical application.^11^ Simplifying the reaction system, it consists of the dehydration reaction of 1,5-AG with free fatty acids (or *N*-fatty acyl-GABA). In particular, free fatty acids are generally inexpensive, available in large quantities, less hazardous, and more stable than fatty acyl chlorides. Therefore, developing a reaction method for dehydration-condensation that simply mixes 1,5-AG and free fatty acids without the use of activating reagents will lead to the most cost-effective method. We have established an inexpensive synthesis method for dehydration-condensation by simple mixing without the use of the organic solvent DMF, expensive fatty acyl chlorides, or expensive activating reagents, and report it here.

## 2. Experimental

### 2.1 Materials and methods

#### 2.1.1 Chemicals

1,5-Anhydro-d-glucitol (1,5-AG) was a high purity product provided by SUNUS Co., Ltd (Kagoshima, Japan). Diisostearyl malate (product name COSMOL™ 222) was purchased from Nisshin OilliO Group, Ltd (Tokyo, Japan). Palmitic acid and stearic acid were provided by Miyoshi Oil & Fat Co., Ltd (Tokyo, Japan). All other reagents and solvents were commercial reagent grade.

#### 2.1.2 TLC analyses

Thin-layer chromatography (TLC) analyses were performed using ethyl acetate (AcOEt)/toluene 1 : 20 as developing solvents and silica gel 60 F_254_ aluminum sheets from Merck Ltd (Tokyo, Japan). The chromogenic reagent was 20% sulfuric acid/ethanol, which was sprayed and heated on a hot plate to develop color. TLC spot density analyses were performed using ImageJ (NIH, Maryland, USA) software after scanning and capturing the TLC as digital images.

#### 2.1.3 Yellowness analyses (whiteness analyses)

The powdered sample was placed into a 2 mL screw-capped glass bottle (*φ* 11.7 mm) until the bottom was no longer visible. The bottom of the glass sample bottle was scanned and captured as a digital image, and the RGB image was converted to an L*a*b* stack using the Lab Stack command in ImageJ software. The b* value was analyzed, and the yellowness of each powder was measured. The closer this value was to 0, the higher the whiteness was judged.

#### 2.1.4 Gel hardness analyses

Gelation was performed using a 2 mL screw-capped glass bottle. Each sample was accurately weighed into this bottle, and in addition the diisostearyl malate we use as a reference oil was accurately weighed into this bottle. This was placed on a hot plate at 100 to 150 °C to dissolve the compounds and then left at room temperature for 12 h to obtain the gels. The gel hardness of the obtained gels was measured using a RHEONER II (RE233005C, Yamaden Co., Ltd, Tokyo, Japan). A 5 mm *φ* spherical plunger was inserted into a gel in a 2 mL screw-capped glass bottle at a speed of 60 mm min^−1^, and the maximum stress up to a 2.5 mm insertion was taken as the gel hardness.

#### 2.1.5 NMR analyses

^1^H-^1^H COSY, HMBC, HSQC, ^1^H NMR, and ^13^C NMR spectra were obtained in CDCl_3_ and D_2_O on Bruker BioSpin spectrometers (AV 400, Bruker Corporation, Madison, MA, USA). Chemical shifts are given in parts per million (ppm) and referenced to Me_4_Si (*δ* 0.00) for CDCl_3_ and tBuOH (*δ* 1.25, 30.29) for D_2_O.

### 2.2 Syntheses

#### 2.2.1 General synthesis method

A total of 6–8 equivalents of palmitic acid or 8 equivalents of stearic acid were added to 1,5-AG (1 g), and the reaction was carried out under a nitrogen atmosphere with or without 0.08 equivalents of base using a Liebig reflux condenser. The cooling water was not circulated in the Liebig reflux condenser because circulating cooling water would cause evaporated palmitic acid to solidify and clog the condenser. The reaction was carried out without the Dean–Stark apparatus because the evaporated palmitic acid solidified and clogged if the Dean–Stark apparatus was installed. Nitrogen was introduced from one outlet of the three-necked flask and released from the top of the Liebig reflux condenser connected to the second outlet to displace the nitrogen in the flask vessel. After sufficient nitrogen replacement, the top outlet was stopped by a glass stopper, and the reaction was performed under positive nitrogen pressure. The reaction temperature was set at 230 °C, and after stirring for the specified reaction time, the heater was turned off, and the reaction mixture was allowed to cool. After cooling sufficiently, 50 mL of acetone was added, and the temperature was raised to the boiling point. Heating was stopped, and the mixture was cooled to room temperature. After crystallization, 50 mL of acetone was added again, and the solidified crystals were stirred at room temperature to break it into powder. The resulting powder was then filtered and washed with acetone to obtain the target organogelator crystals.

## 3. Results and discussion

### 3.1 Synthesis of C16AG

**Fig. 1:**
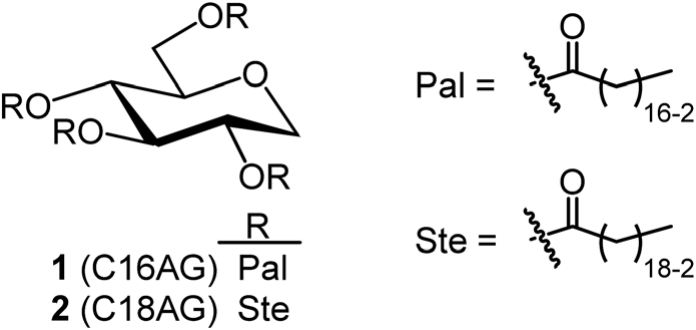
Structures of organogelators C16AG(1) with palmitic acid introduced into 1,5-AG and C18AG(2) with stearic acid.

**Fig. 2:**
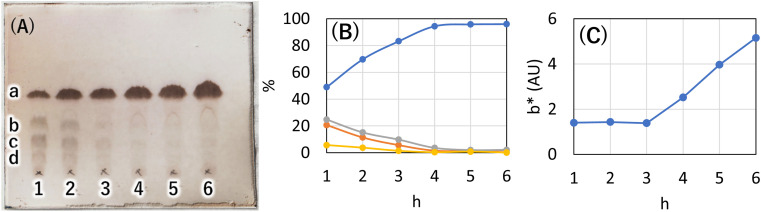
(A) Tracking the reaction through TLC (AcOEt/toluene 1 : 20) when sodium hydroxide was used as catalyst at a reaction temperature of 230 °C. Unit for each number is h. (B) Yield calculated by image analysis of TLC. Image analysis was performed using ImageJ. a: Blue line (C16AG(1), *R*_f_ = 0.52), b: orange line (3-OH-C16AG, *R*_f_ = 0.34), c: gray line (4-OH-C16AG, *R*_f_ = 0.21), d: yellow line (2-OH-C16AG, *R*_f_ = 0.09). (C) The reaction solution sampled at each time point was dried and powdered and placed in a glass bottle, and the bottom of the glass bottle was scanned with a scanner. The scan data was analyzed by ImageJ and the b* values (yellowness) displayed in lab stack were graphed.

Synthetic methods using organic solvents are expensive and furthermore are environmentally hazardous and are not suitable for food applications. Therefore, as a method to esterify hydroxy groups with fatty acids, we searched for an existing technology that performs the esterification conditions without using organic solvents or expensive activated fatty acids. There was a patent for a synthetic route developed for the reaction of polyglycerin with fatty acids at 260 °C using sodium hydroxide as a catalyst (patent JP5727749B2).^13^ In this patent, the ester linkage was formed at high temperatures and without solvents. Assuming that the esterification reaction proceeded similarly with 1,5-AG instead of polyglycerin, we investigated the synthesis of the organogelator 1,5-anhydro-2,3,4,6-tetra-*O*-palmitoyl-d-glucitol (C16AG) (Fig. 1, compound 1). Palmitic acid has a melting point of 61–62 °C and liquifies at higher temperatures. The melting point of 1,5-AG is 142–143 °C. At higher temperatures, both 1,5-AG and palmitic acid are liquid and mix well, and the reaction proceeds efficiently. In addition, 1,5-AG is thermally stable because the most reactive hydroxy group at the 1-position (anomeric position) of glucose is deoxidized. Therefore, 1,5-AG (1.0 g) and palmitic acid (9.4 g) in 1.5 equivalents per hydroxy group of 1,5-AG, for a total of 6 equivalents, and sodium hydroxide (19 mg) in 0.08 equivalents were added, and the reaction proceeded at 160 °C to a uniform liquid mixture. After 4 hours, the progress of the reaction was checked by TLC, and the reaction hardly progressed. The reaction temperature was increased to 185 °C, and after 2.5 hours of reaction, the progress of the reaction was checked by TLC, and a small amount of the target product had formed. After the temperature was further increased to 200 °C for 1.5 hours, the reaction had further advanced. It was confirmed that the esterification reaction proceeded under such basic high temperature conditions. 1,5-AG is a group of compounds cyclized by the dehydration of sorbitol and is a type of sorbitan. One sorbitans is 1,4-anhydro-d-sorbitol, and sorbitan fatty acid esters are well-known surfactants that are generated by esterifying 1,4-anhydro-d-sorbitol with fatty acids. We searched for a method to synthesize sorbitan fatty acid esters and found a patent in which the desired sorbitan fatty acid esters was synthesized by esterifying fatty acids with sorbitol at 230 °C for 2 hours using sodium hydroxide as a catalyst, followed by cyclization with an acid catalyst.^14^ In reference to this method, 8 equivalents of palmitic acid (2 equivalents per hydroxy group) were added to 1,5-AG (1 g), the temperature was fixed at 230 °C, and 0.08 equivalents of sodium hydroxide was used as a catalyst to track the reaction at each hour. A specified amount of 5 M sodium hydroxide solution was added as sodium hydroxide. As shown in Fig. 2(A) and (B), the reaction proceeded in a very good yield, reaching a plateau yield of 94% (the yield was based on TLC image analysis) in 4 hours. The sampled reaction solution was dried and powdered, the powder was scanned, and the b* value was calculated by image analysis to observe yellowing (Fig. 2(C)). The results showed that the reaction solution turned yellow after 4 hours. Based on these results, the optimal reaction temperature was determined to be 230 °C, and the reaction time was 4 hours.

**Fig. 3:**
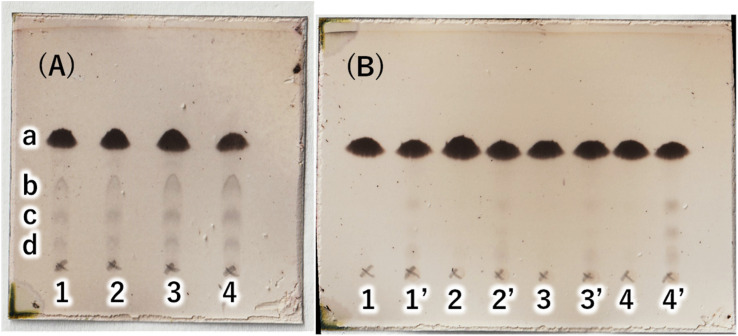
(A) TLC (AcOEt/toluene = 1 : 20) of reaction solutions with each base catalyst. 1: K_2_CO_3_, 2: NaOH, 3: KOH, 4: noncatalyst. a: C16AG (compound 1), yield by image analysis 1: 89%, 2: 86%, 3: 85%, 4: 78%. b: 3-OH-C16AG, c: 4-OH-C16AG, d: 2-OH-C16AG. (B) TLC (AcOEt/toluene = 1 : 20) of primary and secondary crystals obtained in reactions with each base catalyst. Secondary crystals are marked with a dash in each number.

**Fig. 4:**
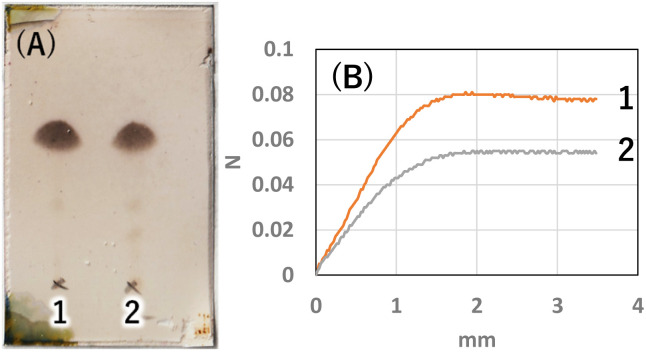
(A) TLC (AcOEt/toluene 1 : 20) of isolated crystals 1: K_2_CO_3_, 2: NaOH. Purity by image analysis 1: 98%, 2: 93%. (B) Gel hardness (1 wt% gel of each sample in diisostearyl malate) 1: 80 mN, 2: 54 mN.

Next, to determine the optimal base, a reaction study with various bases was conducted. The reaction temperature and time were fixed at 230 °C for 4 hours, and 8 equivalents of palmitic acid were added to 1,5-AG (1 g) to compare the effects of various bases. The reaction efficiency was examined by using 0.08 equivalents of potassium carbonate (entry 1), sodium hydroxide (entry 2), and potassium hydroxide (entry 3) as the base. Potassium carbonate was added as a powder, and 5 M aqueous solution was added in specified volumes for sodium hydroxide and potassium hydroxide. The reaction efficiency of entry 4, in which no base was added, was also examined for comparison. The reaction apparatus was the same as described above. After 4 hours of reaction time, heating was stopped, and the reaction solution was cooled to solidify the solution. A small amount of the solidified reaction solution was dissolved in chloroform and confirmed by TLC (AcOEt/toluene = 1 : 20) (Fig. 3(A)). TLC scan data were analyzed by ImageJ, and the yield of target C16AG (compound 1, *R*_f_ value = 0.52, Fig. 3(A), a) was 1: 89%, 2: 86%, 3: 85%, and 4: 78%, respectively. A total of 50 mL acetone was added to these reactants, boiled to dissolve, and then stirred at 50 °C for 12 hours to crystallize. The resulting crystals were quickly filtered at 50 °C to yield 1: 3.69 g (54%), 2: 2.97 g (44%), 3: 1.98 g (29%), and 4: 2.48 g (36%), respectively. The crystals precipitated from the cooled filtrate and were obtained as secondary crystals. The combined isolated yields of the primary and secondary crystals were 1: 90%, 2: 88%, 3: 71%, and 4: 78%, respectively (Table 1). The isolation yields were close to the calculated yields obtained by image analysis in Fig. 3(A). The reaction also proceeded in 78% isolated yield in entry 4 without base. By stirring at 50 °C in acetone, compounds that were not completely reacted and the target C16AG were separated, resulting in the successful isolation of C16AG with a high purity. The primary crystals obtained were of high purity, but TLC of the secondary crystals showed compounds that were not completely reacted (Fig. 3(B)). However, when 1 wt% gel of each sample in diisostearyl malate was used, the secondary crystals showed the same or higher gel hardness than that of the primary crystals, indicating that a purity of approximately 90% is sufficient for use as a gelator (Table 1). The whiteness of the primary crystals was higher in all cases, and crystals with very high whiteness were obtained especially in entry 4, in which no base was used. It was found that for applications requiring whiteness, it is better to perform synthesis without using a base. Based on these results, we decided to change the crystallization conditions from 50 °C to room temperature to obtain crystals with high yield in a single crystallization, as obtaining 100% purity is not necessary. This is especially true for applications as a simple gelling agent that does not require whiteness. Potassium carbonate (entry 1), which produced the highest yield, and sodium hydroxide (entry 2), which produced the second highest yield, were selected as bases for comparison. The reaction was carried out under the same reaction conditions as above, and after 4 hours of reaction time, the heating was stopped, and the reaction solution was cooled and solidified. Then, 50 mL of acetone was added, and the mixture was brought to a boil. Heating was stopped, and the mixture was cooled to room temperature. After solidification, 50 mL of acetone was added again, and the solid was stirred at room temperature to break it into powder. The powder obtained was then filtered and washed with acetone to obtain the target C16AG crystals (Fig. 4(A)). The isolated yield was 94% for entry 1 (potassium carbonate) and 93% for entry 2 (sodium hydroxide), with b* (yellowness) values of 5.0 and 5.2, respectively. Both yield and whiteness were slightly higher for the case of potassium carbonate. Gel hardness was measured (Fig. 4(B)), and it was found that the compound in entry 1 (potassium carbonate) formed a harder gel than the gel prepared with the compound in entry 2 (sodium hydroxide). By comparing the yield, whiteness, and gel hardness, we concluded that potassium carbonate is the best base for C16AG synthesis.

**Table 1:** C16AG synthesis. Yields, purity, gel hardness, and yellowness of each base catalyst used and each number of crystallizations^a^

Entry^b^	Yield^c^ (%)	Purity by TLC^d^ (%)	Hardness^e^ (mN)	Yellowness^f^ (b* value, AU)
1	54	100	32	4.1
1′	36	92	48	5.7
2	44	100	38	4.1
2′	44	92	59	5.3
3	29	100	34	5.4
3′	42	95	60	5.9
4	36	100	41	1.8
4′	42	87	42	4.3

a 1: K_2_CO_3_, 2: NaOH, 3: KOH, 4: noncatalyst.

b Secondary crystals are marked with a dash for each number.

c Isolation yield.

d Purity by image analysis of TLC (Fig. 3(B)).

e Gel hardness (1 wt% gel of each sample in diisostearyl malate).

f The scan data of the bottom of a glass bottle containing powder is analyzed by ImageJ, and the yellowness is analyzed by the b* value displayed on the lab stack on ImageJ. The smaller the b* value, the higher the whiteness.

### 3.2 Synthesis of C18AG

**Fig. 5:**
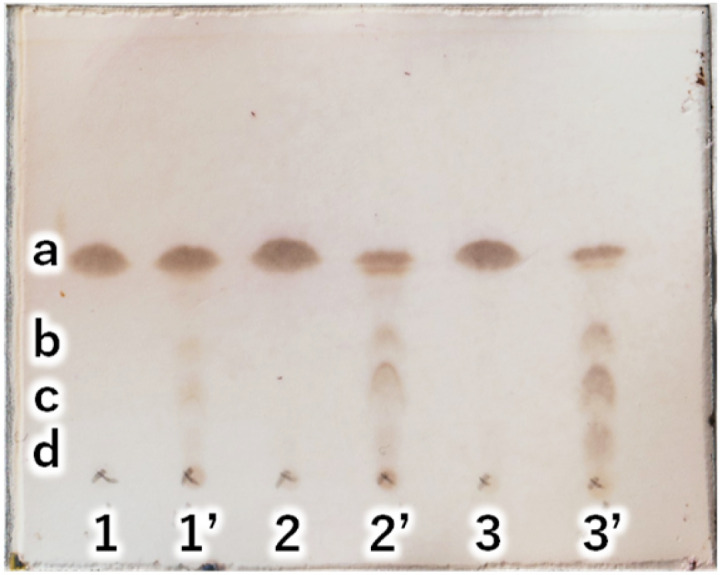
TLC (AcOEt/toluene = 1 : 20) of primary and secondary crystals obtained in the reaction of C18AG (compound 2) synthesis with each base catalyst. Secondary crystals are marked with a dash in each number. 1: K_2_CO_3_, 2: NaOH, 3: noncatalyst. Purity of C18AG (a) by image analysis 1: 100%, 1′: 82%, 2: 100%, 2′: 40%, 3: 100%, 3′: 35%. b: 3-OH-C18AG, c: 4-OH-C18AG, d: 2-OH-C18AG.

As in the synthesis of C16AG, the reaction temperature and reaction time were fixed at 230 °C for 4 hours to investigate the synthesis of organogelator C18AG (Fig. 1, compound 2). Eight equivalents of stearic acid were added to 1,5-AG (1 g) to compare the effects of the two types of bases. Entry 1, potassium carbonate, and entry 2, sodium hydroxide, were each used in 0.08 equivalents to examine the reaction efficiency. Entry 3, without base, was also examined for comparison. The reaction apparatus was the same as above. After 4 hours of reaction time, heating was stopped, and the reaction solution was cooled and solidified. Then, 50 mL of acetone was added and boiled. However, the precipitated crystals did not completely dissolve even when the temperature was increased to boiling. After adding another 50 mL of acetone and stirring at 50 °C for a while, the resulting crystals were quickly filtered at 50 °C to obtain the primary crystals 1: 5.83 g (78%), 2: 6.07 g (81%), and 3: 5.30 g (71%), respectively (Fig. 5 and Table 2). The crystals precipitated from the cooled filtrate and were obtained as secondary crystals. The primary crystals and the secondary crystals together yielded 1: 91%, 2: 91%, and 3: 93%, respectively (Table 2).

**Table 2:** C18AG Synthesis. Yield, purity, gel hardness, and yellowness of each base catalyst used and each number of crystallizations^a^

Entry^b^	Yield^c^ (%)	Purity by TLC^d^ (%)	Hardness^e^ (mN)	Yellowness^f^ (b* value, AU)
1	78	100	39	4.2
1′	13	82	10	6.8
2	81	100	42	4.7
2′	10	40	6	7.9
3	71	100	39	3.5
3′	22	35	6	5.6

a 1: K_2_CO_3_, 2: NaOH, 3: noncatalyst.

b Secondary crystals are marked with a dash for each number.

c Isolation yield.

d Purity by image analysis of TLC (Fig. 5).

e Gel hardness (3 wt% gel of each sample in diisostearyl malate).

f The scan data of the bottom of a glass bottle containing powder is analyzed by ImageJ, and the yellowness is analyzed by the b* value displayed on the lab stack on ImageJ. The smaller the b* value, the higher the whiteness.

Very high purity C18AG could be synthesized as the primary crystal, and the gel hardness was also high (Table 2). Unlike the synthesis of C16AG, the secondary crystal of C18AG had low purity and low gel hardness. In other words, it was found that purification at 50 °C in acetone was necessary to synthesize high purity C18AG. To clarify the reason for this, the solubility of palmitic acid and stearic acid in acetone was tested. The results showed that palmitic acid was soluble in acetone at room temperature, but stearic acid was not. However, stearic acid was found to dissolve when acetone was heated to 50 °C. In other words, stearic acid dissolves and is separated at 50 °C, the condition for primary crystallization of C18AG, but at room temperature, the condition for secondary crystallization, stearic acid crystallizes and mixes with secondary crystals of C18AG. In addition, compounds that were not fully reacted during synthesis were present in C18AG, and the percentage of these compounds in the secondary crystals was relatively increased. These factors may have contributed to the decrease in gel hardness. As observed in the C16AG synthesis, the whiteness of the compound without base catalyst was the highest in the C18AG synthesis (Table 2, entry 3). Although only primary crystals can be used in the synthesis of C18AG and the yield is lower than in the synthesis of C16AG, the reaction without base catalyst is optimal for gelling agents that require whiteness. For gelator applications that do not require whiteness, the yields, gel hardness, and whiteness were similar for both potassium carbonate and sodium hydroxide, indicating that both bases can be used.

### 3.3 Synthesis of C_16_GABA-AG

**Fig. 6:**
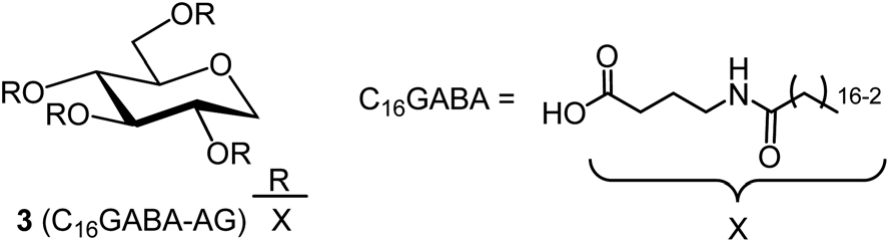
Structure of organogelator C_16_GABA-AG(3), a fatty acid with an amide group attached to 1,5-AG.

We investigated the synthesis of organogelator C_16_GABA-AG (Fig. 6, compound 3), which is 1,5-AG with C_16_GABA, a fatty acid bonded to γ-aminobutyric acid (GABA) by an amide bond. The coupling of GABA and palmitic acid was performed by known methods to synthesize C_16_GABA.^12^ The synthesis of C_16_GABA-AG (compound 3) was investigated by fixing the reaction temperature and reaction time at 230 °C for 4 hours, as in the synthesis of C16AG and C18AG. The reaction efficiency was examined by adding 8 equivalents of C_16_GABA (6.66 g) to 1,5-AG (0.4 g) and 0.08 equivalents of potassium carbonate, which has provided good results thus far, as a base (entry 1). For comparison, the reaction efficiency without base was also examined (entry 2). The reaction apparatus was the same as above. In contrast to previous results, the coloration of the reaction solution progressed as the reaction temperature increased, and by the time it reached 230 °C, the solution turned dark black. This coloration proceeded regardless of the presence or absence of base. After 4 hours of reaction time, the heating was stopped, and the reaction solution was cooled and solidified, dissolved in heated acetone as previously described, and powdered at room temperature. The resulting powder was filtered and washed with acetone to give a black solid (1: 2.03 g, 2: 1.61 g). NMR measurements of the obtained solids revealed that the target C_16_GABA-AG (compound 3) was not present in both entries 1 and 2, and that the main component of each solid was C16AG (compound 1) (ESI Fig. 11s†). In other words, the nitrogen atom of the intramolecular amide group nucleophilically attacked the GABA-derived carbonyl carbon activated by high temperature, and after intramolecular cyclization, the amide bond was cleaved, and free palmitic acid was released. The free palmitic acid was ester bonded with 1,5-AG under high temperature conditions, and C16AG (compound 1) was synthesized. Therefore, we considered introducing GABA and fatty acids to 1,5-AG separately in two steps. The reaction was carried out without base by adding 8 equivalents of GABA (5.0 g) to 1,5-AG (1 g). The reaction apparatus was the same as above. In contrast to previous results, a large number of bubbles were generated when the reaction temperature reached approximately 150 °C, but by the time the temperature reached 230 °C, bubbles were no longer generated. After 4 hours of reaction time, the heating was stopped, and the liquid was cooled, but the reaction liquid did not solidify and remained liquid even at room temperature. NMR measurements of the liquid showed that 1,5-AG did not react and that 2-pyrrolidone, an intramolecularly cyclized GABA, was quantitatively synthesized (ESI Fig. 12s†). In other words, as discussed above, the dehydration reaction was found to proceed preferentially by intramolecular cyclization of GABA. Unfortunately, we have not succeeded in synthesizing C_16_GABA-AG at high temperature in a solvent-free manner.

## 4. Conclusions

We have successfully synthesized C16AG and C18AG, organogelators, which contain a starch-derived cyclic alcohol bonded with a fatty acid, without using any organic solvents or activated fatty acids in the reaction system. This has established the basic technology for industrial mass synthesis. After the reaction, the gelators needed to be crystallized to improve purity, and acetone was used only at this time. Since acetone can be easily distilled and purified, and can be reused, we assume that it will not be a bottleneck in industrial production. 1,5-AG is a compound in which the most reactive hydroxy group at the 1-position (anomeric position) of glucose, which constitutes starch, is deoxidized. Glucose usually melts and thermally decomposes in the temperature range of about 150 °C (ref. 15) and is not suitable for reactions at higher temperatures. However, 1,5-AG is very thermally stable and can withstand reaction conditions of 230 °C due to the absence of the hydroxy group at 1-position, the most reactive group of glucose. The gelators synthesized here have the ability to gel diisostearyl malate, which we used as a reference oil. The gelators were also shown to be synthesizable without using a base, yielding whiter crystals than those obtained when a base was used. It was found that potassium carbonate was superior base in terms of yield, whiteness, and gel hardness when synthesized using a base. We plan to use this method to perform industrial mass synthesis in the future.

## Conflicts of interest

There are no conflicts to declare.

## Supplementary Material

RA-013-D3RA01328F-s001
