# Supplementary material for: High-temperature solvent-free synthesis of low-molecular-weight organogelators consisting of starch-derived 1,5-anhydro-d-glucitol coupled with fatty acids
Source: RSC Adv. 2023 Mar 21;13(14):9316–21. doi: 10.1039/d3ra01328f (PMC10029127; doi:10.1039/d3ra01328f)
Supplement: RA-013-D3RA01328F-s001 [file RA-013-D3RA01328F-s001.pdf]

Electronic Supplementary Material (ESI) for RSC Advances.
This journal is © The Royal Society of Chemistry 2023

Supporting information

High-temperature solvent-free synthesis of low-molecular-weight organogelators consisting of starch-derived 1,5-anhydro-D-glucitol coupled with fatty acids

Shiro Komba* and Rika Iwaura

Food Research Institute, National Agriculture and Food Research Organization, 2-1-12, Kannondai, Tsukuba, Ibaraki 305-8642, Japan.

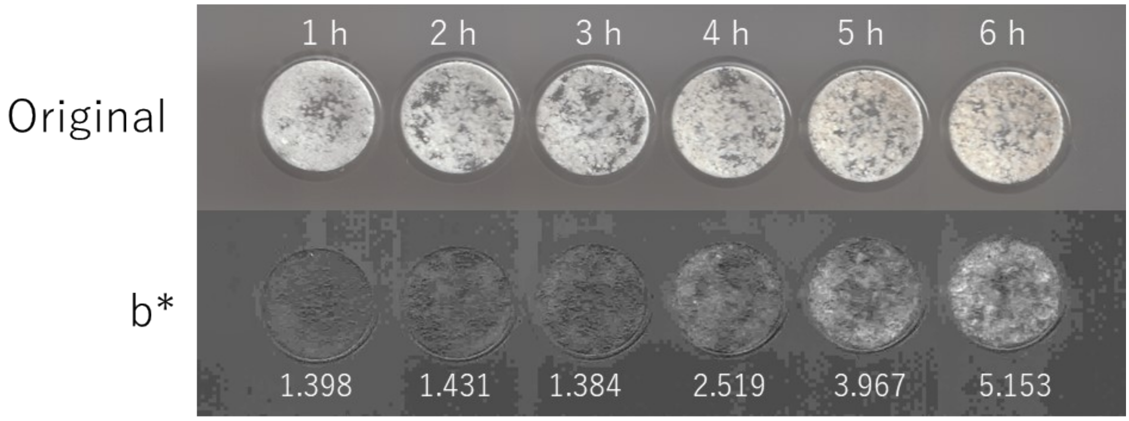

Fig. 1s Scan image and converted b* stack image for measuring yellowness (whiteness) shown in (C) of Fig. 2.

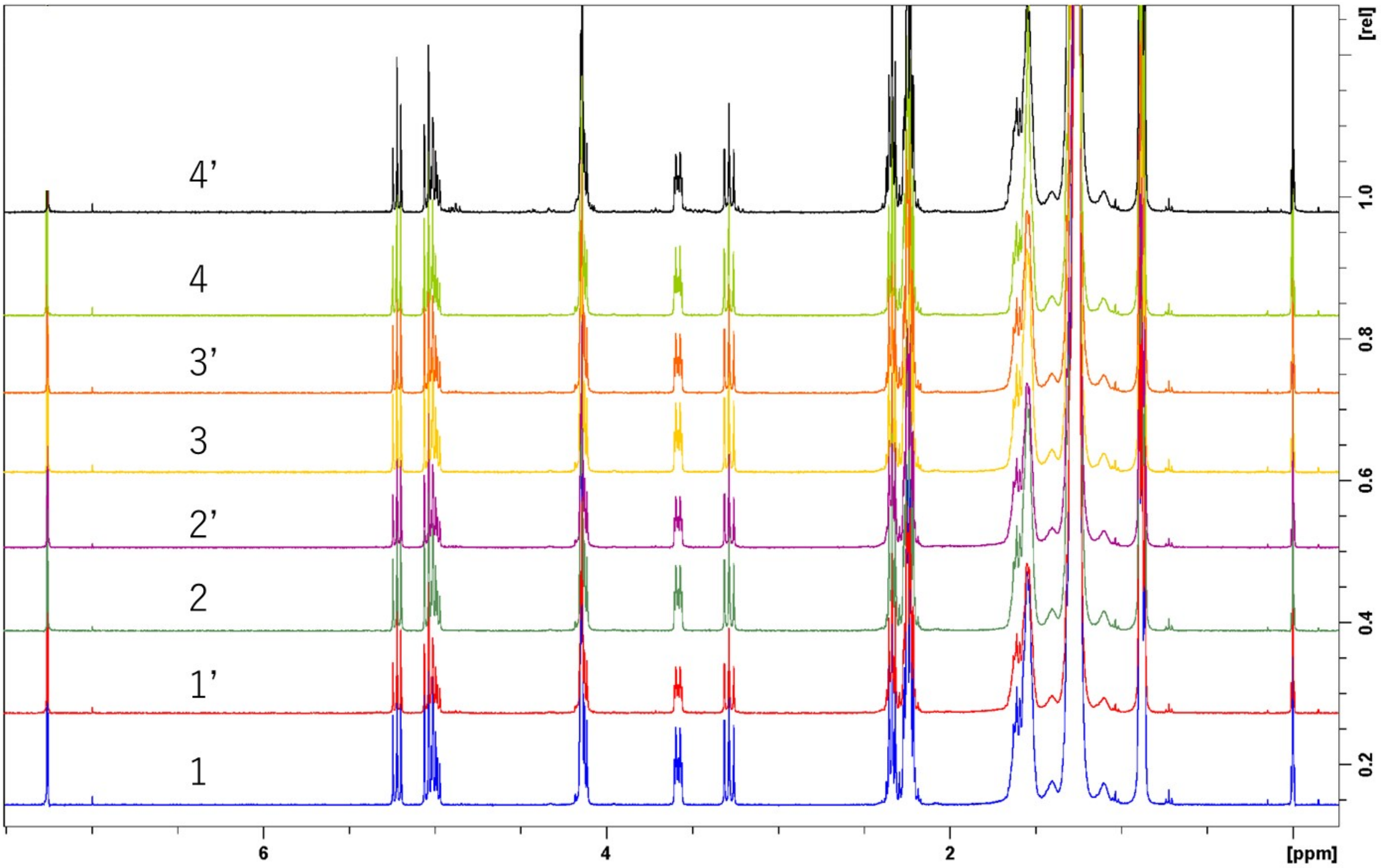

Fig. 2s ^{1}H-NMR spectra of each sample of C16AG in $CDCl_3$ (Me_4Si = 0 ppm). 1: K_2CO_3, primary crystal (Table 1, entry 1), 1': K_2CO_3, secondary crystal (Table 1, entry 1'), 2: NaOH, primary crystal (Table 1, entry 2), 2': NaOH, secondary crystal (Table 1, entry 2'), 3: KOH, primary crystal (Table 1, entry 3), 3': KOH, secondary crystal (Table 1, entry 3'), 4: noncatalyst, primary crystal (Table 1, entry 4), 4': noncatalyst, secondary crystal (Table 1, entry 4').

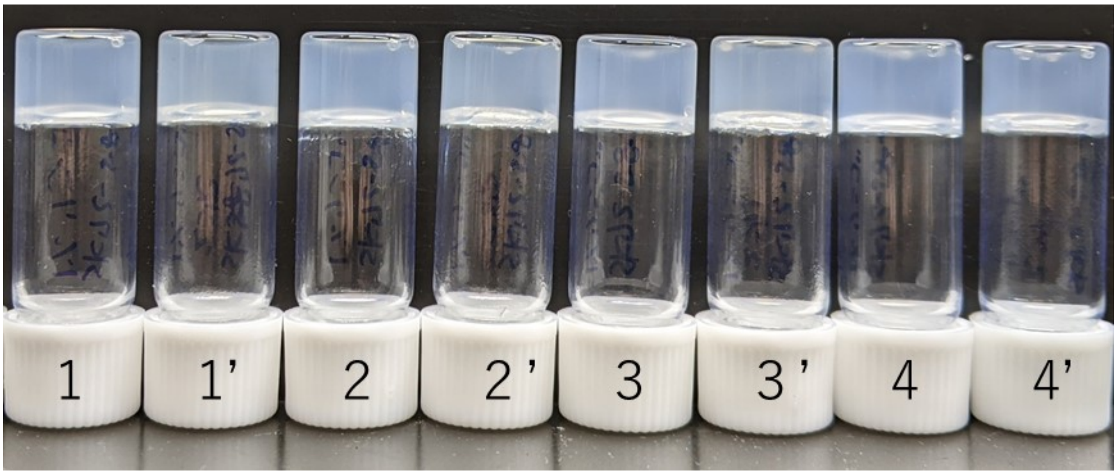

Fig. 3s Appearance of gels (1 wt % gel of each sample in diisostearyl malate) in each entry shown in Table 1. None of the gels flowed down when inverted.

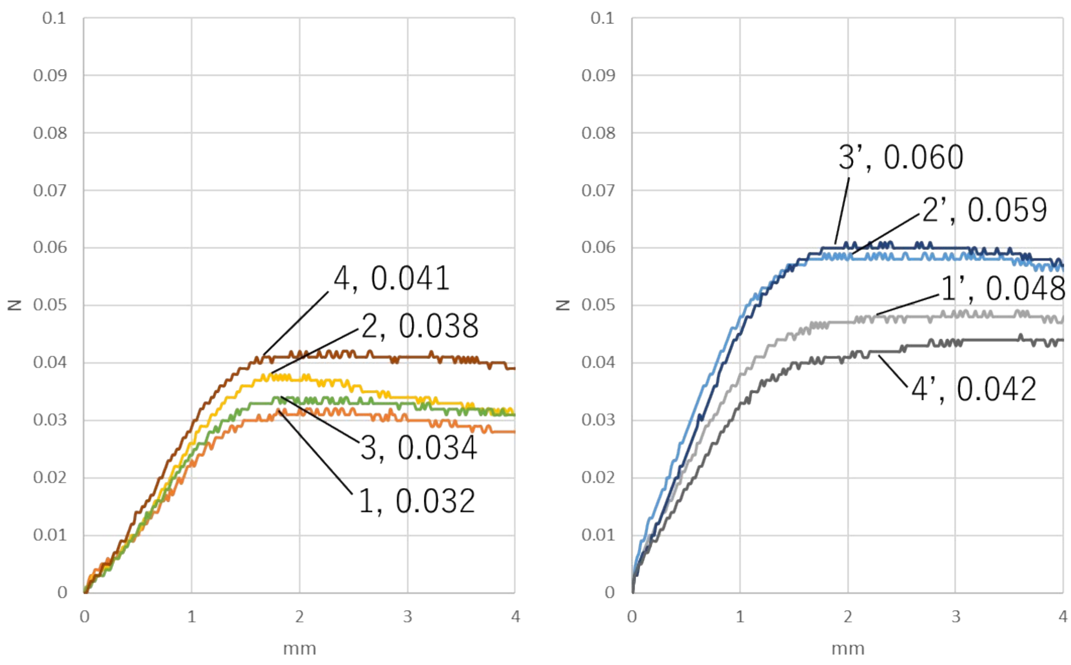

Fig. 4s Gel hardness measurement results for each entry are shown in Table 1.

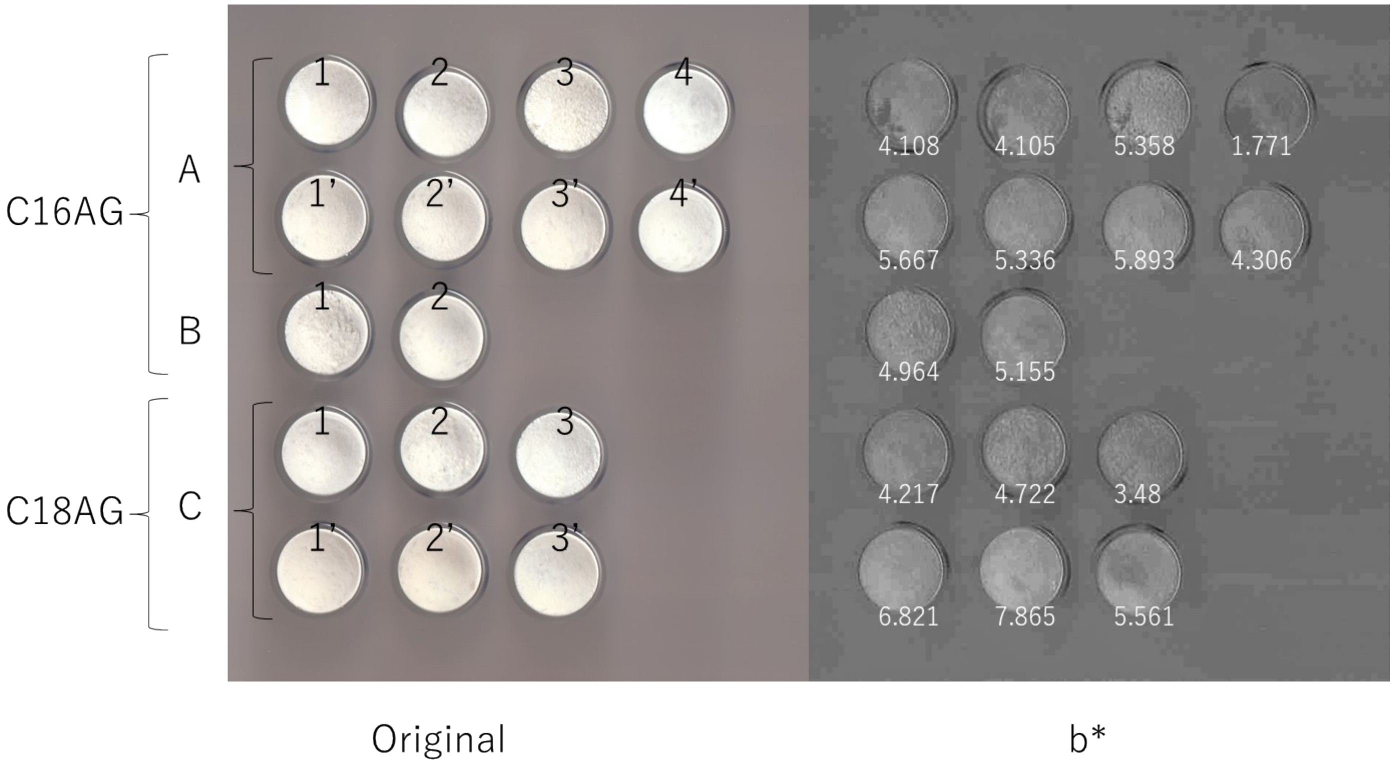

Fig. 5s The scan image and converted b* stack image of each sample. A: Scan image and converted b* stack image for measuring yellowness (whiteness) shown in Table 1. B: Scan image and converted b* stack image for measuring the yellowness (whiteness) of the compounds shown in Fig. 4. C: Scan image and converted b* stack image for measuring yellowness (whiteness) shown in Table 2.

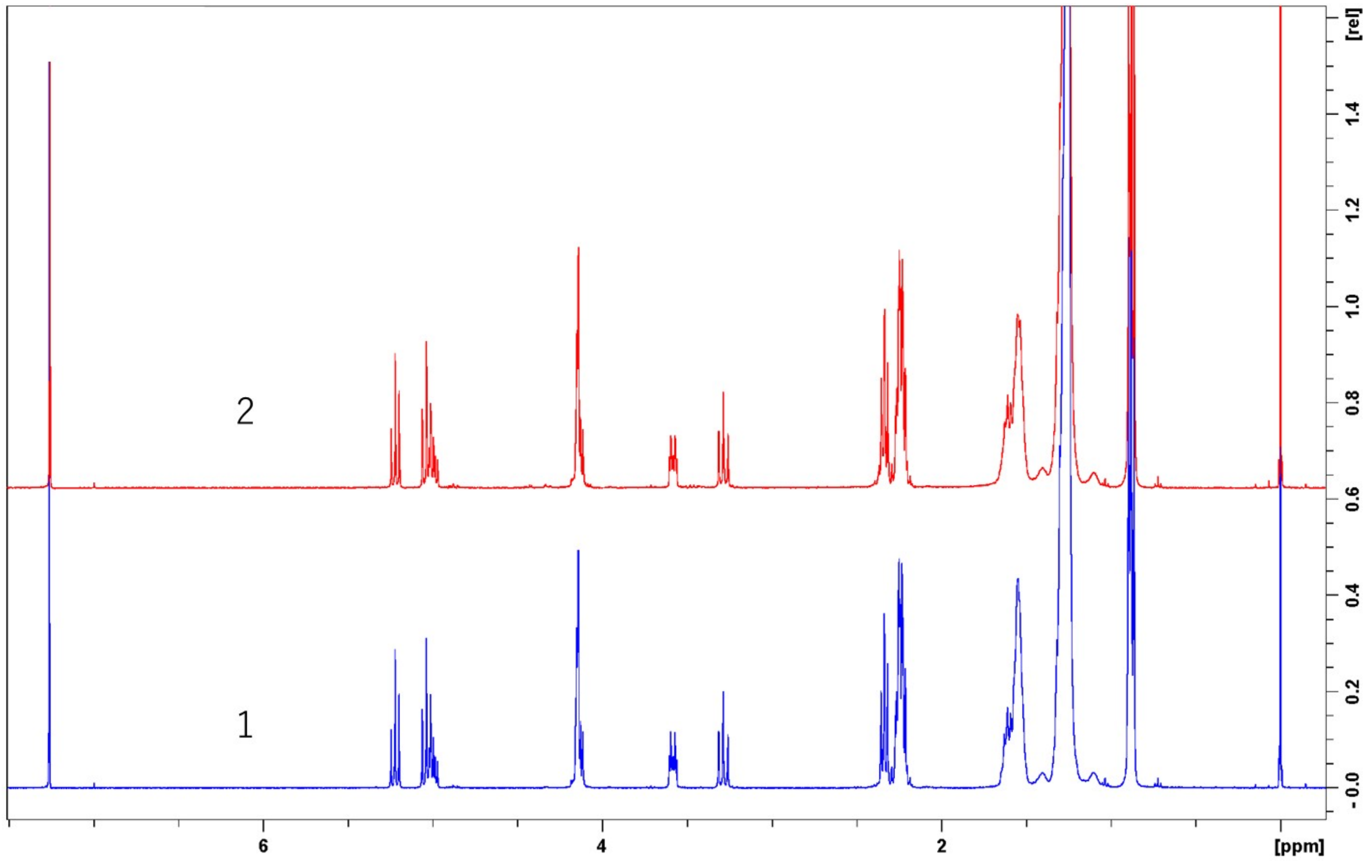

Fig. 6s ^{1}H-NMR spectra of each sample of C16AG in $CDCl_3$ (Me_4Si = 0 ppm). 1: K_2CO_3, (Fig. 4, entry 1), 2: NaOH, (Fig. 4, entry 2).

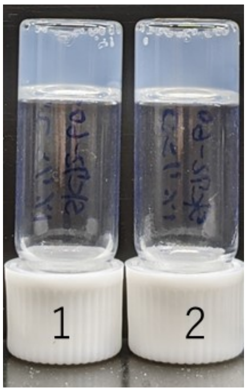

Fig. 7s Appearance of gels (1 wt % gel of each sample in diisostearyl malate) in both entries shown in Fig. 4. Both gels did not flow down when inverted.

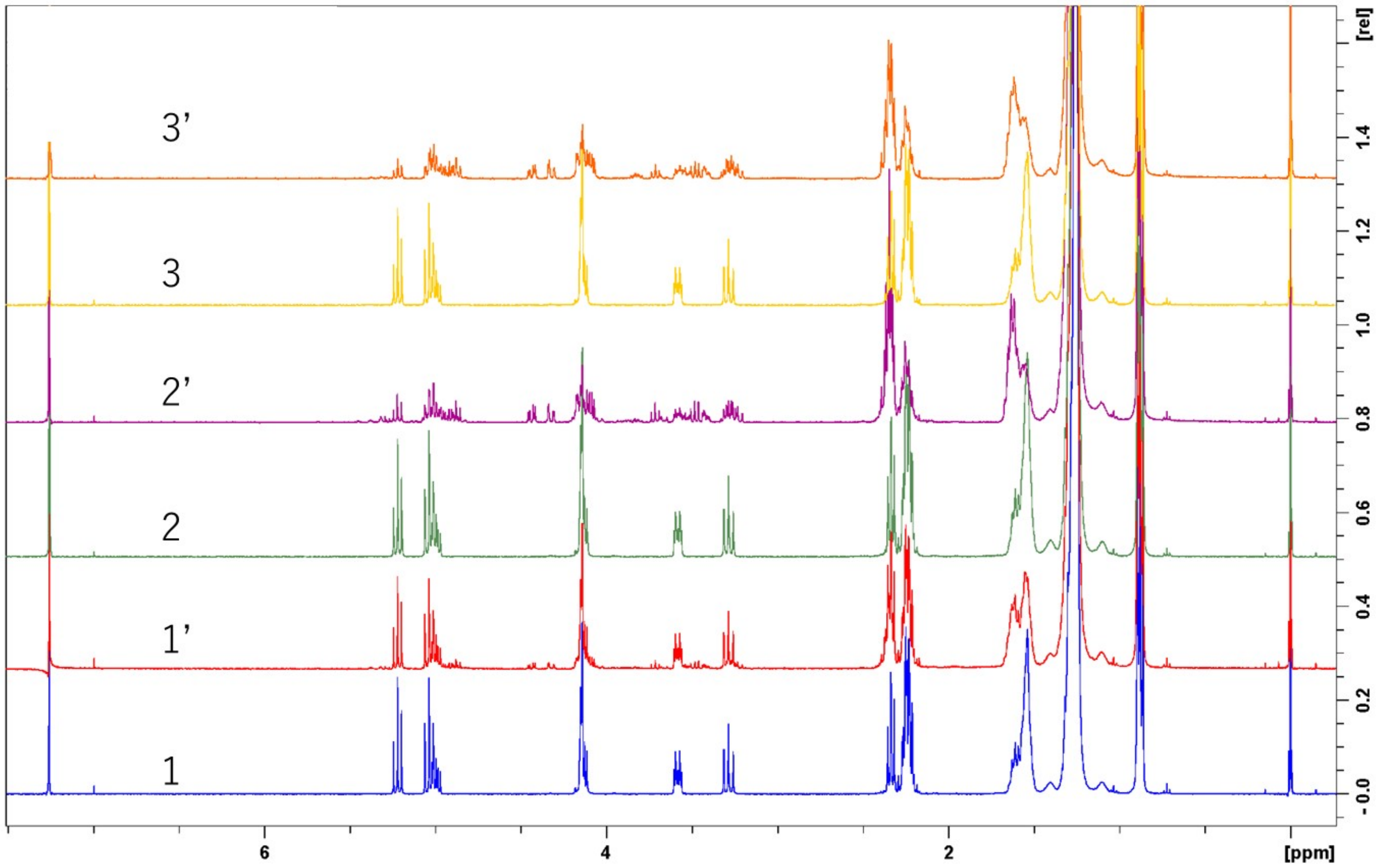

Fig. 8s ^{1}H-NMR spectra of each sample of C18AG in $CDCl_3$ (Me_4Si = 0 ppm). 1: K_2CO_3, primary crystal (Table 2, entry 1), 1': K_2CO_3, secondary crystal (Table 2, entry 1'), 2: NaOH, primary crystal (Table 2, entry 2), 2': NaOH, secondary crystal (Table 2, entry 2'), 3: noncatalyst, primary crystal (Table 2, entry 3), 3': noncatalyst, secondary crystal (Table 2, entry 3').

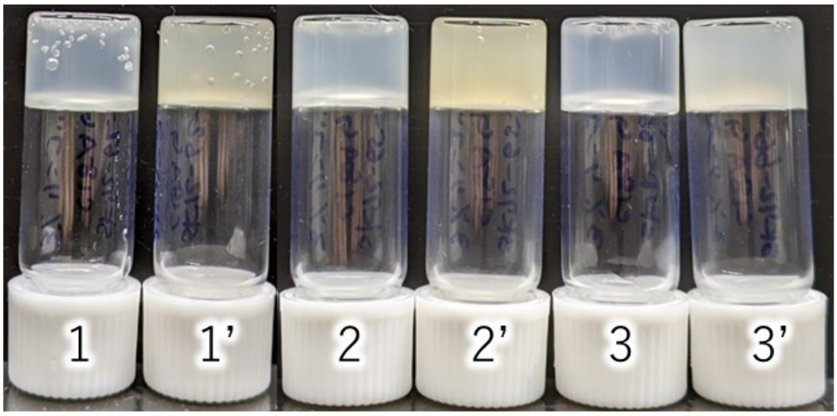

Fig. 9s Appearance of the gels (3 wt % gel of each sample in diisostearyl malate) in each entry shown in Table 2. None of the gels flowed down when inverted.

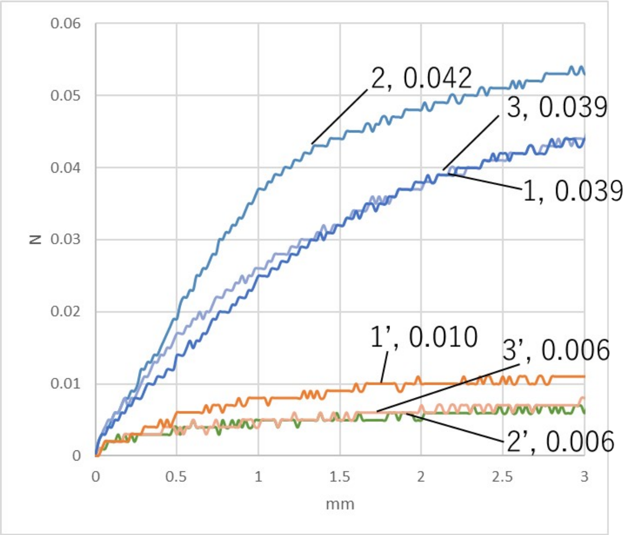

Fig. 10s Gel hardness measurement results for each entry are shown in Table 2.

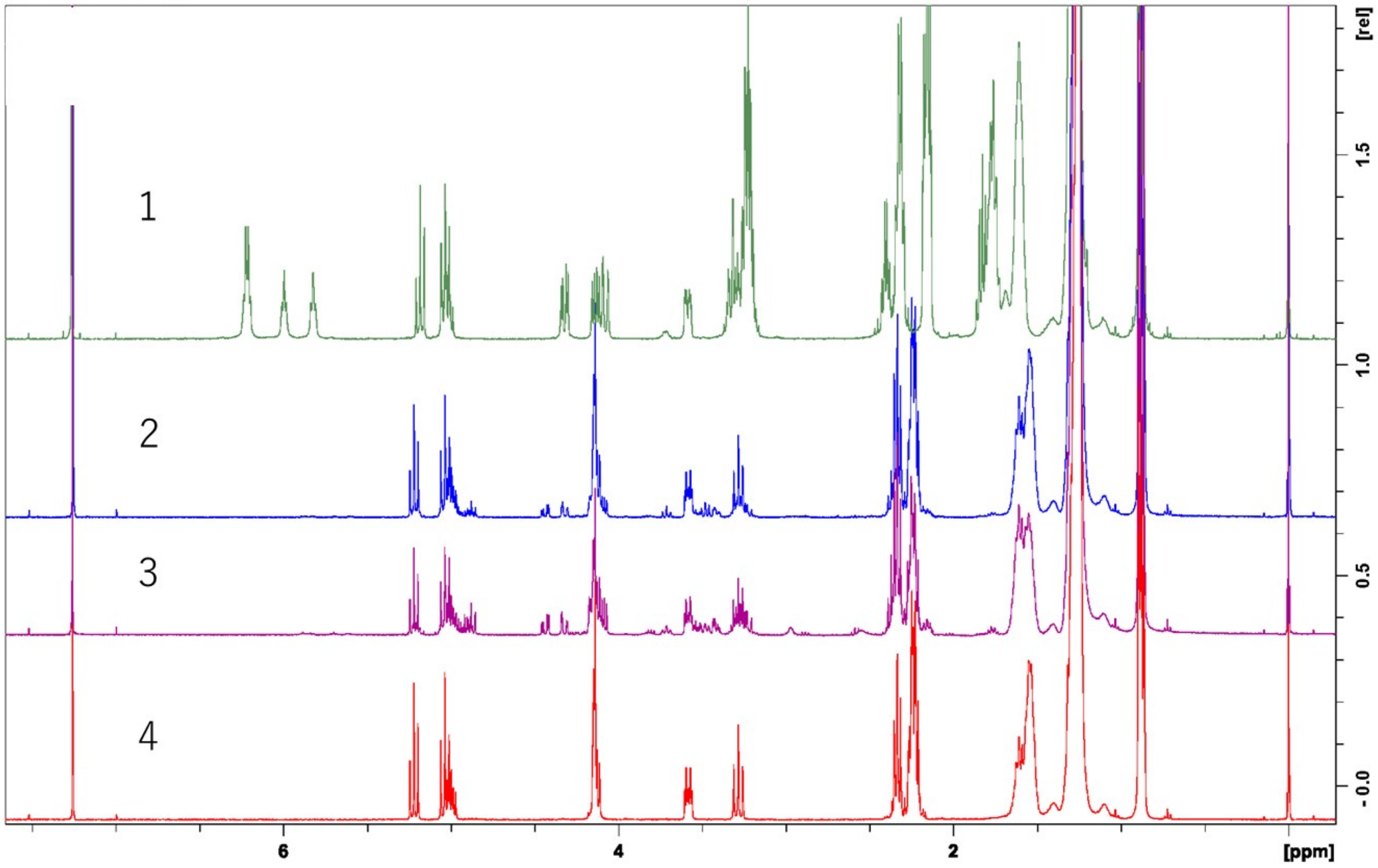

Fig. 11s ^{1}H-NMR spectra of each sample in $CDCl_3$ (Me_4Si = 0 ppm). 1: C_{16}GABA-AG standard, 2: Reaction products of 1,5-AG and C_{16}GABA by thermal synthesis (with base catalyst), 3: Reaction products of 1,5-AG and C_{16}GABA by thermal synthesis (without base catalyst), 4: C16AG standard. In both entries 2 and 3, C16AG was synthesized instead of C_{16}GABA-AG.

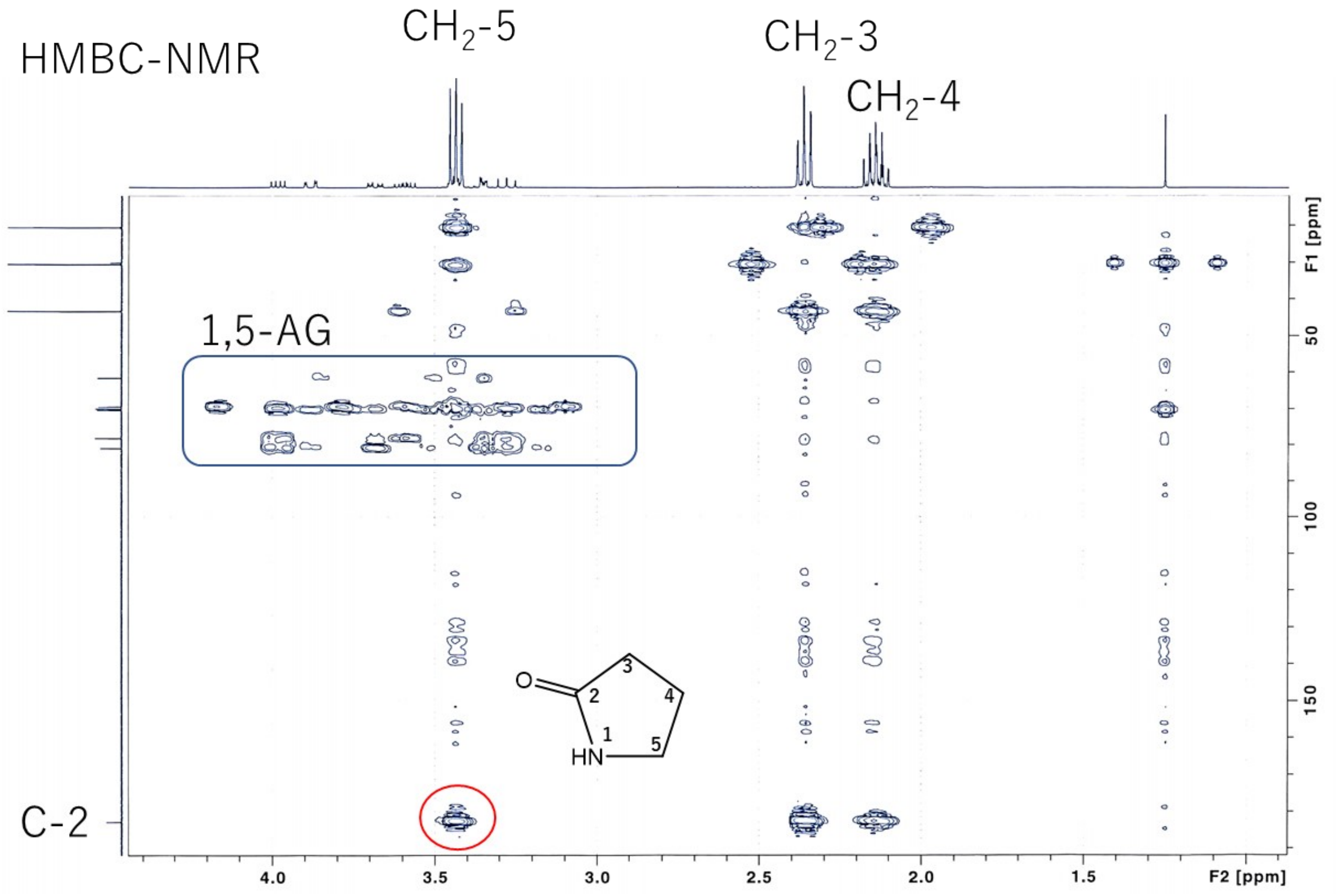

Fig. 12s HMBC-NMR spectra of the reaction product of 1,5-AG and GABA by thermal synthesis in D_2O (*t*BuOH = 1.25 ppm, 30.29 ppm). The cross peaks of CH_2-5 and C-2 indicate intramolecular cyclization.
